# Time outdoors positively associates with academic performance: a school-based study with objective monitoring of outdoor time

**DOI:** 10.1186/s12889-023-15532-y

**Published:** 2023-04-04

**Authors:** Jingjing Wang, Padmaja Sankaridurg, Thomas Naduvilath, Wayne Li, Ian G. Morgan, Kathryn A. Rose, Rebecca Weng, Xun Xu, Xiangui He

**Affiliations:** 1 Shanghai Eye Disease Prevention and Treatment Center, Shanghai Eye Hospital, Shanghai Vision Health Center & Shanghai Children Myopia Institute, Shanghai, 200030 China; 2 Brien Holden Vision Institute, Sydney, Australia; 3 School of Optometry and Vision Science, University of New South Wales, Sydney, Australia; 4 Division of Biochemistry and Molecular Biology, Research School of Biology, Australian National University, Canberra, ACT Australia; 5 Discipline of Orthoptics, Graduate School of Health, University of Technology Sydney, Ultimo, NSW Australia; 6 Department of Ophthalmology, National Clinical Research Center for Eye Diseases, Center of Eye Shanghai Key Laboratory of Ocular Fundus Diseases, Shanghai Engineering Center for Visual Science and Photomedicine, Shanghai General Hospital, Shanghai Jiao Tong University, Shanghai, 200080 China

**Keywords:** Academic performance, Children, Myopia, Outdoor time

## Abstract

**Background:**

To explore the relationship between outdoor time and academic performance among school-aged children.

**Methods:**

This study was designed as a cross-sectional study. Data were derived from a school-based prospective children myopia intervention study (STORM). Outdoor time was recorded by self-developed algorithm-validated wristwatches in real-time and calculated as the cumulative average of 10 months. The academic performance was recorded and provided by the participating schools and further standardized. Other information was collected using an online standardized questionnaire. Mixed-effects model and B-Spline method were used to investigate the association between time spent on different types of daily activity, including outdoor activity and academic performance.

**Results:**

A total of 3291 children with mean age 9.25 years were included in the final analysis. Overall, outdoor time was associated with academic performance in a non-linear manner; specifically, not exceeding 2.3 h per day, outdoor time was positively associated with academic performance; exceeding 2.3 h per day, this association became non-significant. Likewise, daily sleep duration and out-of-school learning time were associated with academic performance in a non-linear manner, resulting in turning points of 11.3 and 1.4 h per day, respectively. Separate analysis showed that outdoor time and sleep duration but not out-of-school learning time were positively associated with academic performance in Chinese, mathematics and English.

**Conclusion:**

Outdoor time, sleep duration and out-of-school learning time were associated with academic performance in a non-linear manner. Promotion of outdoor time may not negatively impact on academic performance.

**Trial registration:**

Our study was registered in ClinicalTrials.gov (Identifier: NCT02980445).

**Supplementary Information:**

The online version contains supplementary material available at 10.1186/s12889-023-15532-y.

## Introduction

Outdoor activity is reportedto be associated with several positive physical and mental health outcomes among children. Specifically, previous studies reported that onset of myopia was associated with a combination of genetic and environmental factors [1], of which, improving outdoor time by 2 h per day has the benefit of reducing the risk by nearly 50%. This reduced risk is attributed to the intensity of light levels outdoors rather than physical activity or sport [2–4]. The other beneficial effects of outdoor time are well recognized; outdoor time and activity reduces the incidence of obesity, depression, and improves cognitive and mental health abilities [5–8].

In spite of the overwhelming evidence in support of outdoor time, children in countries with a high prevalence of myopia lag their counterparts from other countries concerning outdoor time. A survey conducted in China found that the majority of students spent less than 2 h of outdoor time per day; nearly 35.3% spent < 1 h per day [9]. Reported reasons for lack of or reduced outdoor time were safety concerns [10], a greater interest in indoor screen-based activities [11] and concerns related to darkening of the skin due to sun exposure and prioritization of academic achievements [12].

Time spent outdoors was closely related to the academic burden. Chinese parents conventionally hold the view that outdoor activities would reduce the time available for learning and thus result in poor academic performance. Furthermore, Chinese schools always put restrictions on students’ outdoor time arising from the class break and physical education class for the sake of promotion of academic performance. Importantly, to date and to the best of our knowledge, there has been no data indicating if outdoor time would adversely influence the academic performance among school-aged children. In the present study, using data collected from an intervention trial aiming at assessing the relation between outdoor time and myopia, we explored the relationship between outdoor time and academic performance among school-aged children.

## Material and methods

### Study design and study population

Data for the current study were gathered from a school-based, prospective study (Shanghai Time Outside to Reduce Myopia (STORM)) conducted in Shanghai, China. In the STORM study, 16 districts of Shanghai were divided into cities and suburbs according to their geographical location and economic conditions. 8 districts were selected by stratified sampling according to population proportion and then 3 schools were randomly selected from each districts. Finally, 6259 children aged 6 ~ 9 years were enrolled from 24 primary schools utilizing this cluster-based sampling technique. The STORM study was conducted from 2016 to 2018 and the methodology of the study was reported previously [13]. The STORM study followed the tenets of the Declaration of Helsinki for experimentation on Humans and was approved by the Ethics Committee of Shanghai General Hospital. Informed consent was obtained from the parents/caregivers of all participants enrolled in the STORM study.

### Data collection

#### Outdoor time

The STORM study was two years in duration. During the second year, outdoor time was objectively collected using wearable smart wristwatches for 10 months from March to December 2018. Data were not gathered during January and February 2018 as this period coincided with winter vacation. All participants were required to wear the wristwatches during daytime (7 a.m. to 7 p.m.) from March (the beginning of spring semester of 2018) to December 2018. Data on luminance, ultraviolet light, step count and weather condition were collected with the wristwatches and transmitted to a computer terminal every minute. Only data were included in the analysis from children who accumulatively wore the wristwatches more than 5 h per day and more than 120 days in total.

#### Academic performance

Data on academic performance from the fall semester of 2018 were provided by the participating schools and used in this study. Three scoring methods for AP were applied by the schools in the study: a) grading using an alphabetical system (A to D); b) character grades (evaluated as “excellent, good, medium or poor”); and specific numerical scores. First, all grading was transformed to a numerical system of 1 to 4; second, min–max normalization rescaling method was adopted to unify the numerical grading into an interval between 0 and 1([0,1]); the last, all rescaled scores were ranked within classes to record the percentile ranking as the academic performance included in the final analysis. Comprehensive academic performance was computed as the average percentile rankings of disciplines of Chinese, mathematics and English.

#### Questionnaire

Parents/caregivers were required to fill an online questionnaire using an APP. The questionnaire included basic information (age, gender, parental education and family monthly income) as well as time spent on various kinds of daily activity by children, including time spent sleeping, time spent learning out of school on weekend days, statutory holidays and weekdays. The questionnaire was administered every semester of 2018 (spring and fall semesters) and at the end of the summer vacation of 2018. A question asking about out-of-school tutoring time per day was added for the summer questionnaire. The sleep duration and out-of-school learning time (OSLT) were defined as a weighted value according to calendar days:

1 $$OSLT\ (hours\ per\ day) = (learning\ time\ per\ day\ on\ weekend\ days/statutory\ holidays * 76\ days + learning\ time\ per\ day\ in\ weekdays * 168\ days+ learning\ time\ per\ day\ in\ summer\ vacations * 62\ days)/306\ days.$$

The number of 76, 168 and 62 in Eq. 1 resulted from the fact that there were 76 weekend days/statutory holidays, 168 weekdays and 62 days in summer vacations during the study period. The number of 306 was the total of abovementioned numbers.

2 $$Sleep\ duration\ per\ day\ (hours\ per\ day) = (Sleep\ duration\ per\ day\ on\ weekend\ days/statutory\ holidays * 76\ days + Sleep\ duration\ per\ day\ in\ weekdays * 168 days\ + Sleep\ duration\ per\ day\ in\ summer\ vacations * 62 days)/306\ days.$$

The numbers in Eq. 2 were defined as were in Eq. 1.

### Statistical analysis

Base on the data of luminance, ultraviolet light, step count and weather condition gathered from the wristwatches, real-time discrimination of indoor versus outdoor environmental state was performed using a support vector machine (SVM) algorithm. The method has been validated and published elsewhere [14]. According to the judgment of indoor versus outdoor, the daily outdoor time can be obtained by accumulating the minutes marked as outdoor every day. The real-time wristwatch wearing status were also recorded.

SAS 9.4 (SAS Institute, Cary, NC, USA) and GraphPad Prism (GraphPad Software, San Diego, California USA) were utilized for data cleaning and analysis. Variables’ distributions were examined by Kolmogorov–Smirnov test. Continuous variables with normal distribution or approximate normal distribution were presented as means ± standard deviation (SD), those with non-normal distribution were presented as median with quantiles, and categorical data were shown as rates (proportions). A Chi-square test was used for comparison of categorical data. A t-test or variance analysis was used for comparison of continuous data. A two-sided *p* < 0.05 was considered statistically significant. A mixed-effects model was performed to investigate the effect of outdoor time, sleep duration and OSLT on academic performance with adjusting for possible confounding factors and the cluster effects. The possible interactions between variables were also analyzed. Further, a B-Spline method with knots of 3–5 was used to fit the curve between outdoor time, sleep duration, OSLT, and the possible thresholds values were explored. *P* values < 0.05 were considered as statistically significant.

## Results

### General characteristics

Of the 3442 participants, 151(4.4%) were excluded as the information on academic performance was not available. No significant differences in sex and age distributions were observed between the included and excluded participants. (3291 versus 151; age groups: χ^2^ = 0.320, *p* = 0.852; gender: χ^2^ = 0.023, *p* = 0.879).

The mean (SD) age was 9.3(0.6) years and 48.6% were boys. The mean (SD) outdoor time, sleep duration, OSLT per day were 2.0(0.5), 9.5(0.5) and 2.4(1.1) hours, respectively. Additionally, the mean (SD) tutoring time in summer vacation were 3.3 ± 4.0 h. Overall, boys spent more time outdoors and slept less compared to girls, but gender differences were not observed in OSLT and tutoring time in summer vacation. Concerning Chinese, English and comprehensive academic performance, girls scored better than boys (*P* < 0.05), but no gender difference was observed in AP of mathematics (*p* = 0.161). Details are presented in Table 1.

**Table 1:** The general characteristics by gender of the participants

Variables	Total (*n* = 3291)	Girls (*n* = 1692)	Boys (*n* = 1599)	P
Mean ± SD				
BMI, kg/m^2^	17.84 ± 3.02	17.26 ± 2.58	18.45 ± 3.31	< 0.001
Outdoor time, hrs/day	2.02 ± 0.50	1.95 ± 0.48	2.10 ± 0.50	< 0.001
Sleep duration, hrs/day	9.53 ± 0.48	9.55 ± 0.48	9.50 ± 0.48	0.008
Out-of-school learning time, hrs/day	2.43 ± 1.06	2.43 ± 1.06	2.43 ± 1.06	0.870
Extracurricular class in summer vacation, hrs/week	3.26 ± 4.04	3.28 ± 4.04	3.23 ± 4.03	0.606
Comprehensive academic performance	54.17 ± 27.32	55.77 ± 26.96	52.48 ± 27.60	0.001
Chinese lesson	53.48 ± 25.91	56.19 ± 25.46	50.63 ± 26.07	< 0.001
Mathematics lessons	53.20 ± 25.62	52.56 ± 25.35	53.87 ± 25.88	0.161
English lessons	53.65 ± 24.59	55.83 ± 23.80	51.34 ± 25.21	< 0.001
n(%)				
Age				
≤ 8 yrs	1249 (38.0)	630 (37.2)	619 (38.7)	0.509
9 yrs	1598 (48.6)	838 (49.5)	760 (47.5)	
≥ 10 yrs	444 (13.5)	224 (13.2)	220 (13.8)	
Father education				
Junior high school and below	819 (24.9)	403 (23.8)	416 (26.0)	0.543
High school or vocational school	993 (30.2)	521 (30.8)	472 (29.5)	
Undergraduate or junior college	1282 (39.0)	665 (39.3)	617 (38.6)	
Master degree or above	90 (2.7)	46 (2.7)	44 (2.8)	
Mother education				
Junior high school and below	1024 (31.1)	496 (29.3)	528 (33.0)	0.113
High school or vocational school	884 (26.9)	459 (27.1)	425 (26.6)	
Undergraduate or junior college	1235 (37.5)	657 (38.8)	578 (36.1)	
Master degree or above	47 (1.4)	27 (1.6)	20 (1.3)	
Family income (RMB/month)				
< = 6000	835 (25.4)	415 (24.5)	420 (26.3)	0.567
6000–10,000	1109 (33.7)	579 (34.2)	530 (33.1)	
10,000–20,000	883 (26.8)	446 (26.4)	437 (27.3)	
> 20,000	383 (11.6)	204 (12.1)	179 (11.2)	

*Abbreviation*: *BMI* Body mass index, *hrs* Hours, *SD* Standard deviation

### Factors correlated with Academic performance

Regression analysis showed that outdoor time, sleep duration and OSLT were significantly correlated with CAP (outdoor time: estimate = 0.054, *p* = 0.002; sleep duration: estimate = 0.099, *p* < 0.001; OSLT: estimate = -0.078, *p* < 0.001).

**Fig. 1:**
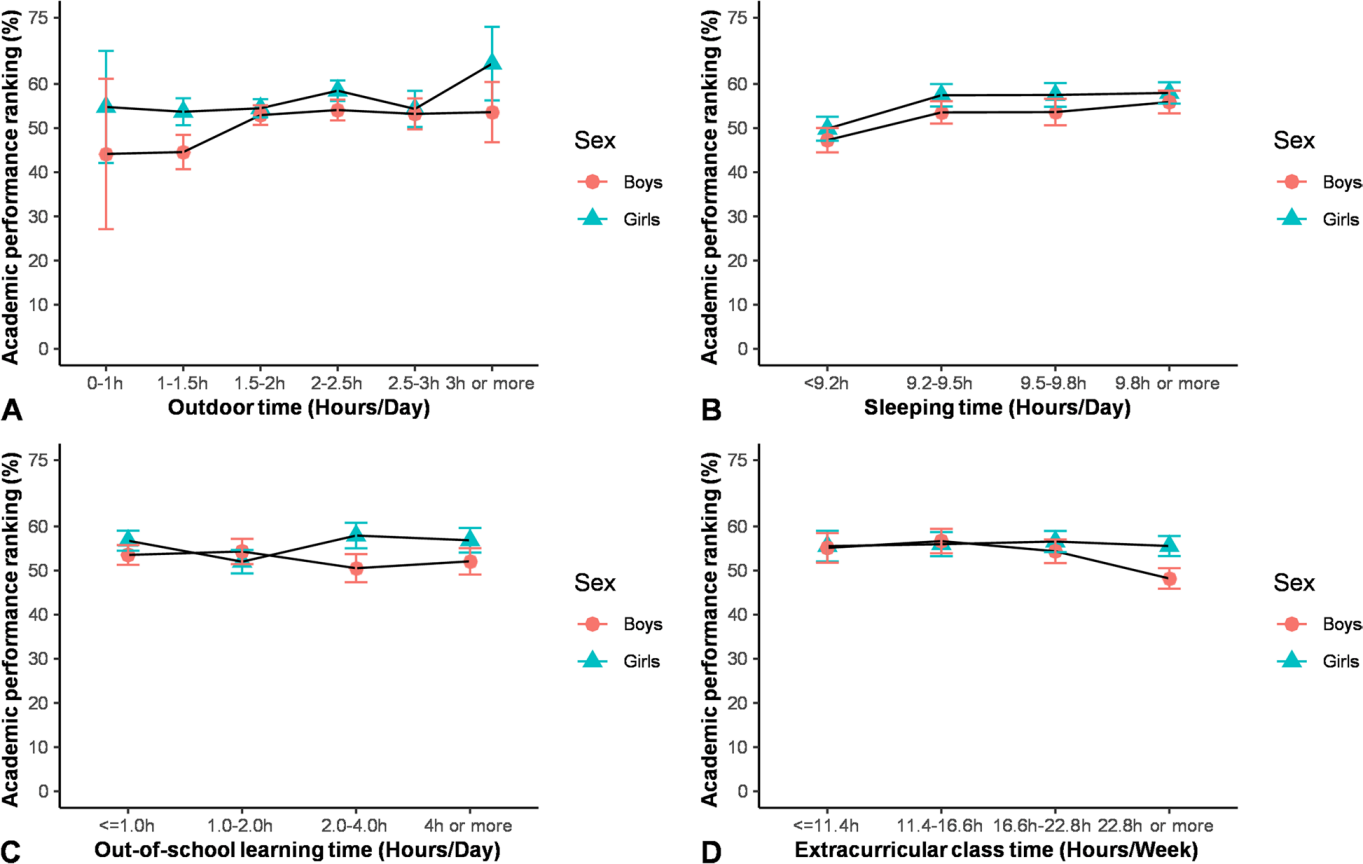
The Association between comprehensive academic performance and related factors. These four plots shows the academic performance ranking means and standard deviations of participants with different (**A**) Outdoor time, **B** Sleep duration, **C** Out-of-school learning time and (**D**) Extracurricular class in summer vacation

No difference was found between tutoring activities in summer and academic performance (estimate = -0.011, *p* = 0.533) (Fig. 1).

**Fig. 2:**
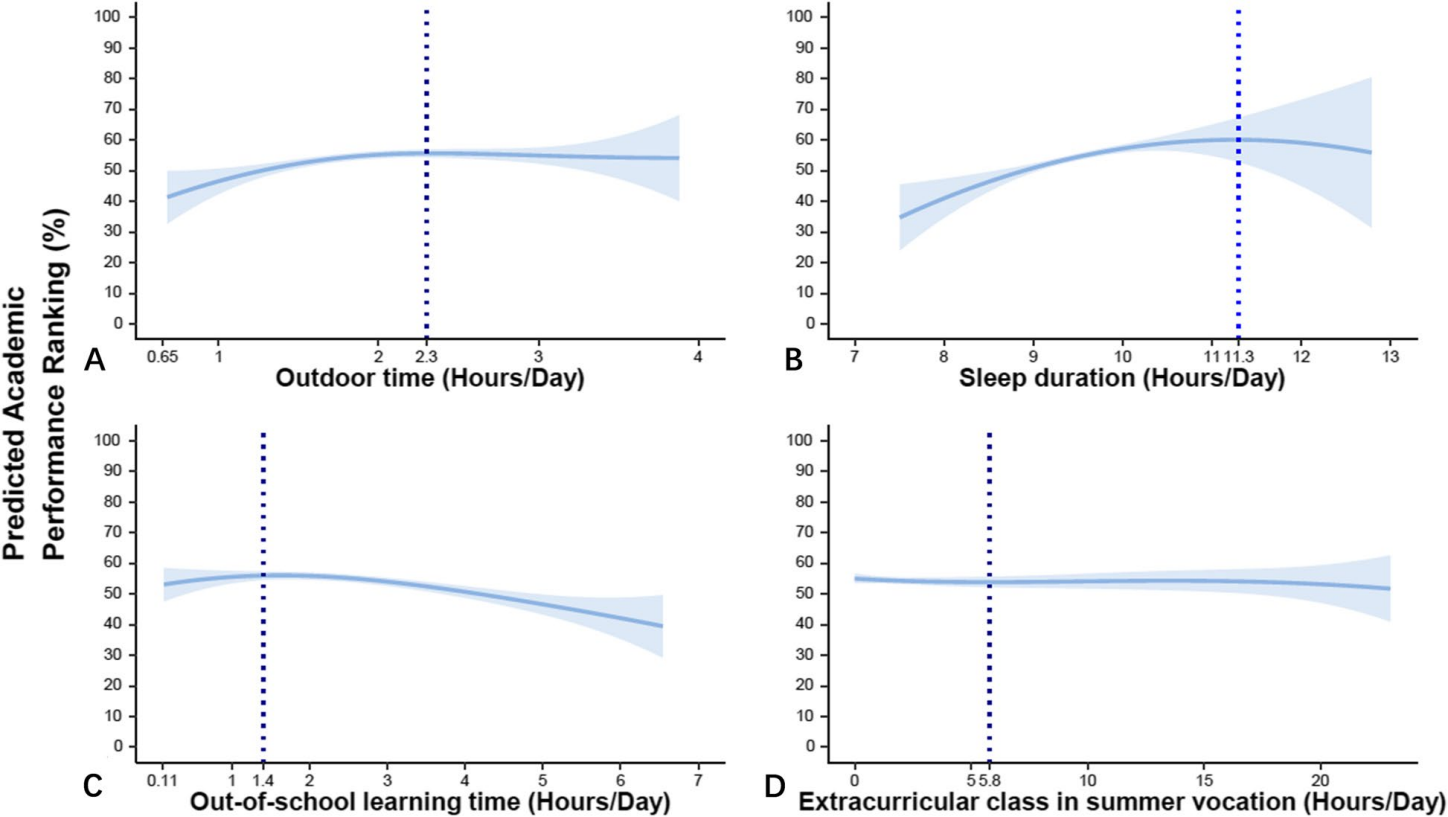
Non-linear association between comprehensive academic performance and time spent on different types of daily activity modeled using the P-Spline method. The fitted curves of academic performance ranking against (**A**) Outdoor time, **B** Sleep duration, **C** Out-of-school learning time and (**D**) Extracurricular class are shown in red and their 95% prediction interval are shown in blue

Further explorations of the relationship were conducted using P-spline (penalized B-spline) method (Fig. 2). Academic performance increased with outdoor time but reached a plateau with outdoor time > 2.3 h/day. Similarly, academic performance increased with sleep duration and OSLT, but reached inflection point at 11.3 h/day and 1.4 h/day, after which the relationships turned to be negative.

### Multivariate analysis on factors associated with comprehensive academic performance

Multivariate analysis found more outdoor time and sleep duration and less OSLT were associated with greater academic performance (all *p* < 0.001, outdoor time *β* = 6.494, sleep duration *β* = 5.865, OSLT *β* = -1.800). Girls and older age tended to achieve better academic performance (gender: *β* = 3.445, *p* < 0.001; age: *β* = 2.540, *p* = 0.002). As for parental factors, children’s academic performance was positively associated with their parents’ educational level and family income generally (Table 2).

**Table 2:** Linear mixed effect model of factor associated with academic performance (stratified by outdoor time)

Variables	Total population		Outdoor time ≤ 2.3 h/day		Outdoor time > 2.3 h/day	
	β (95%CI)	*P*	β (95%CI)	*P*	β (95%CI)	*P*
Outdoor time, hrs/day	6.494(4.148 ~ 8.840)	< 0.001	10.126(6.308 ~ 13.945)	< 0.001	1.022(-5.702 ~ 7.745)	0.766
Sleeping time, hrs/day	5.865(3.873 ~ 7.856)	< 0.001	5.690(3.430 ~ 7.949)	< 0.001	6.460(2.266 ~ 10.653)	0.003
Learning time out of school, hrs/day	-1.800(-2.700 ~ -0.900)	< 0.001	-1.375(-2.418 ~ -0.333)	0.010	-2.803(-4.603 ~ -1.003)	0.002
Gender, girls/boys	3.445(1.543 ~ 5.348)	< 0.001	3.289(1.078 ~ 5.500)	0.004	4.527(0.770 ~ 8.283)	0.018
Age	2.540(0.945 ~ 4.135)	0.002	2.509(0.649 ~ 4.369)	0.008	3.125(-0.022 ~ 6.273)	0.052
Father education						
Junior high school and below	—		—		—	
High school or vocational school	3.645(0.722 ~ 6.567)	0.015	3.407(-0.167 ~ 6.980)	0.062	4.036(-1.094 ~ 9.165)	0.123
Undergraduate or junior college	7.847(4.329 ~ 11.365)	< 0.001	8.343(4.180 ~ 12.506)	< 0.001	5.240(-1.468 ~ 11.949)	0.126
Master degree or above	9.122(2.003 ~ 16.241)	0.012	8.582(0.623 ~ 16.542)	0.035	6.938(-9.973 ~ 23.849)	0.421
Mother education						
Junior high school and below	—		—		—	
High school or vocational school	1.376(-1.510 ~ 4.261)	0.350	1.742(-1.729 ~ 5.213)	0.325	0.874(-4.383 ~ 6.131)	0.744
Undergraduate or junior college	5.932(2.525 ~ 9.338)	0.001	6.644(2.650 ~ 10.637)	0.001	-2.538(-24.239 ~ 19.164)	0.819
Master degree or above	9.776(0.614 ~ 18.937)	0.037	12.407(2.227 ~ 22.586)	0.017	2.987(-3.551 ~ 9.524)	0.37
Family income (RMB/month)						
< = 6000	—		—		—	
6000–10,000	1.596(-0.909 ~ 4.100)	0.212	2.729(-0.264 ~ 5.723)	0.074	-0.633(-5.256 ~ 3.990)	0.788
10,000–20,000	4.359(1.544 ~ 7.175)	0.002*	4.935(1.626 ~ 8.244)	0.004*	4.037(-1.425 ~ 9.500)	0.147
> 20,000	3.987(0.385 ~ 7.590)	0.030*	4.830(0.760 ~ 8.900)	0.020*	2.051(-5.921 ~ 10.024)	0.614

No interaction was found between outdoor time and other possible factors: gender, *P* = 0.923; father education, *P* = 0.377; mother education, *P* = 0.178

When stratified by the threshold of 2.3 h of outdoor time per day, the effect of outdoor time, age, parental education and family income on academic performance was significant in those with time spent outdoors < 2.3 h/day, but not relevant in those with > 2.3 h/day (Table 2).

### Further analysis on factors associated with academic performance in different disciplines

Outdoor time and sleep duration were positively associated with academic performance in Chinese (outdoor time: *β* = 5.770, *p* < 0.001; sleep duration: *β* = 4.682, *p* < 0.001), mathematics (outdoor time: *β* = 4.051, *p* < 0.001; sleep duration: *β* = 3.302, *P* = 0.001) and English (outdoor time: *β* = 5.737, *P* < 0.001; sleep duration: *β* = 5.569, *P* < 0.001), whereas OSLT was negatively associated (Chinese *β* = -1.921, *P* < 0.001, mathematics*β* = -1.814, *P* < 0.001, English *β* = -1.041, *P* = 0.016). Older age and a higher level of parental education were associated with better performance, and girls showed better performance in Chinese and English than boys the multivariate model (Table 3).

**Table 3:** Linear mixed effect model of factor associated with academic performance of different disciplines

Variables	Chinese lesson		Mathematics lessons		English lessons	
	β (95%CI)	*P*	β (95%CI)	*P*	β (95%CI)	*P*
Outdoor time, hrs/day	5.770(3.476 ~ 8.064)	< 0.001*	4.051(1.851 ~ 6.252)	< 0.001*	5.737(3.534 ~ 7.940)	< 0.001*
Sleeping time, hrs/day	4.682(2.667 ~ 6.698)	< 0.001*	3.302(1.296 ~ 5.309)	0.001*	5.569(3.682 ~ 7.457)	< 0.001*
Learning time out of school, hrs/day	-1.921(-2.827 ~ -1.015)	< 0.001*	-1.814(-2.710 ~ -0.918)	< 0.001*	-1.041(-1.884 ~ -0.198)	0.016*
Gender, girls/boys	6.101(4.190 ~ 8.013)	< 0.001*	-1.379(-3.284 ~ 0.526)	0.156	4.716(2.923 ~ 6.509)	< 0.001*
Age	2.831(1.245 ~ 4.416)	0.001*	1.816(0.230 ~ 3.402)	0.025*	1.599(0.109 ~ 3.09)	0.036*
Father education						
Junior high school and below	—		—		—	
High school or vocational school	2.349(-0.620 ~ 5.318)	0.121	1.254(-1.715 ~ 4.224)	0.408	3.153(0.362 ~ 5.944)	0.027*
Undergraduate or junior college	5.040(1.498 ~ 8.582)	0.005*	4.191(0.666 ~ 7.715)	0.020*	6.532(3.216 ~ 9.848)	< 0.001*
Master degree or above	3.391(-3.769 ~ 10.550)	0.353	7.336(0.186 ~ 14.487)	0.044*	9.275(2.551 ~ 15.999)	0.007*
Mother education						
Junior high school and below	—		—		—	
High school or vocational school	1.726(-1.187 ~ 4.639)	0.245	2.309(-0.599 ~ 5.217)	0.120	0.692(-2.043 ~ 3.426)	0.620
Undergraduate or junior college	4.941(1.551 ~ 8.331)	0.004*	4.021(0.652 ~ 7.389)	0.019*	6.580(3.393 ~ 9.766)	< 0.001*
Master degree or above	10.166(1.108 ~ 19.225)	0.028*	7.337(-1.754 ~ 16.427)	0.114	6.421(-2.134 ~ 14.976)	0.141
Family income (RMB/month)						
< = 6000	—		—		—	
6000–10,000	-0.383(-2.927 ~ 2.162)	0.768	-0.002(-2.545 ~ 2.542)	0.999	0.553(-1.838 ~ 2.944)	0.650
10,000–20,000	2.741(-0.081 ~ 5.562)	0.057	0.604(-2.203 ~ 3.412)	0.673	1.194(-1.450 ~ 3.837)	0.376
> 20,000	2.660(-0.944 ~ 6.264)	0.148	1.371(-2.206 ~ 4.947)	0.453	0.061(-3.306 ~ 3.427)	0.972

^*^P < 0.05

## Discussion

This study was, to the best of our knowledge, the first to explore the relationship between outdoor time and academic performance based on objective outdoor data. The study findings revealed that when not exceeding a cumulative total of 2.3 h per day, outdoor time was positively associated with academic performance; however, when exceeding a cumulative total of 2.3 h per day, outdoor time was not correlated with academic performance. This finding inferred that more time spent outdoors would not negatively impact on academic performance. Also, the study findings showed that specific sleep duration and OSLT had positive influences on academic performance.

Interestingly, several studies found that physical activity (PA) was effective in improving children and adolescents’ academic performance [15–20], especially in mathematics. Specifically, a two-year follow-up study from Finland found that higher level of fitness(aerobic fitness, muscular fitness and motor skills) was correlate with better grade point average(GPA) of adolescents [18]. Similarly, a trial from Sydney found that moderate-to-vigorous PA intervention had a positive effect on mathematics performance in adolescents [19]. Another study integrated PA into the teaching of mathematics and language lessons in elementary schools children for a two-year period and found that the mathematics and spelling performance of children improved over the two years [20]. These studies, however, did not differentiate the effect of indoor versus outdoor activities, just focusing on activity categories and intensity.

Concerning myopia prevention, studies revealed that outdoor time rather than sports activities played a vital role in preventing myopia [2, 4]. In our study, the effect of outdoor time on academic performance was higher than that reported in the abovementioned studies, which were similar to ours in terms of design and statistical methods and had good comparability. Therefore, we speculate that outdoor time may be more effective than the physical exercise itself in improving academic performance. Moreover, at the practical level, outdoor time rather than physical activities do not emphasize on physical load and technical sports, therefore it has the merits of being safer, few qualification requirements for school teachers and better operation.

To the best of our knowledge, the relationship between objectively measured outdoor time and academic performance among children has not been reported so far. However, previous experiments indicated that light could change the arousal mechanism through neurohormone, which in turn may improve children's attention in class [21]. This may help to explain the internal mechanism of the relationship between outdoor time and academic achievements. Furthermore, the nature of light dependence is similar to the mechanism of preventing myopia by outdoor activities. To date, several high-quality intervention studies have confirmed that outdoor time can effectively prevent myopia [22–25]. The main hypothesis was that the exposure of natural light promotes from the retina the release of dopamine which was a known eye growth inhibitor and thus prevents myopia [2, 4, 26]. Although more researches are needed to clarify the above mechanisms, they may serve as a potential common basis for promoting outdoor activities to improve learning performance and prevent myopia.

Due to the negative impact of excess outdoor time on the academic achievement of children and other concerns among stakeholders, including parents and teachers, the outdoor time among children is restrained. This situation is especially prominent in countries with a high prevalence of myopia. According to previous reports, the average outdoor time in 12 countries (Australia, Brazil, Canada, China, Colombia, Finland, India, Kenya, Portugal, South Africa, the UK and the USA) evaluated by questionnaire was 2.52 h per day, while there were only 1.05 h per day in Chinese senior high school students [27, 28]. When monitored by objective device, daily outdoor time among Australian children and Singapore children was 1.75 h per day and 1.02 h per day, respectively [29]. The findings from our study that not exceeding 2.3 h per day the outdoor time could play a positive role in increasing academic performance may serve as evidence of promoting the children’s outdoor time.

Our previous meta-analysis showed that two hours of outdoor activities per day could reduce the risk of myopia by 50% and three hours could reduce by 75% [3]. Combined with the finding of 2.3-h threshold in this paper, we suggest that efforts should be made to promote daily outdoor time at least to 2 h and would be better to 3 h since more outdoor time will not reduce academic performance.

It was also found that appropriate sleep duration could promote academic performance, which was consistent with previous studies [30, 31]. Interestingly, in the present study, the inflection point appeared on 11.3 h per day, indicating that exceeding 11.3 h per day, the sleep duration was not significantly associated with academic performance. A possible explanation is that excessively long sleep duration could take up the learning time and thus reduce knowledge acquisition.

In addition, the relationship between OSLT and academic achievements did not show a simple positive correlation. Only within 1.4 h per day, OSLT was positively associated with academic performance, while, it was negatively correlated with the improvement of academic performance when exceeding 1.4 h/day. Besides, extracurricular classes in summer vacation, which is very common in urban areas of China, could not improve academic performance. All these messages could inform school administrators and parents of adjusting their learning arrangements for their students or their kids.

There were several limitations to the study. Firstly, the effect of outdoor time and physical exercises on academic performance was not analyzed separately, which did not allow us to confirm whether the effect of outdoor time was caused by physical exercises or outside itself. Currently, we are working on this topic and hopefully will obtain both types of data and publish in the near future. Secondly, the academic performance was not evaluated by a unified test, but collected from self-assessment results completed by the schools. We, however, have conducted standardization in class level, which would avoid part of the information bias caused by the schools’ desire to obtain positive results when using a unified test. Last but not the least, it was a cross-sectional designed analysis. In order to better evaluate the impact of outdoor time over a period of time, the variable of outdoor time was calculated as the cumulative average of 10 months based on data from the wristwatch which we regarded was more objective and accurate than the traditional questionnaire. Our follow-up study would provide an opportunity to explore the causal correlation between the outdoor time and academic performance.

## Conclusion

In conclusion, the results of the present study provided new information and understanding of the educational institutions and parents that increased outdoor time may not have a negative impact on academic performance. On the contrary, excess OSLT may negatively impact academic performance. Since there was a certain concern that outdoor activities would have a negative impact on academic performance, which is an obstacle factor to improve children's outdoor time. The findings from this study are expected to improve perception as well as behavior among related stakeholders, including parents and teachers, and to open up a new insight for children myopia intervention practice and academic achievement as well as other children related health issues.

## Supplementary Information

**Additional file 1: Appendix 1.** Information about the wearable device. **Appendix 2.** Algorithm of outdoor/indoor discrimination. **Appendix 3.** Compliance.

## Data Availability

The datasets used and/or analysed during the current study are available from the corresponding author on reasonable request.
