# Supplementary material for: Time outdoors positively associates with academic performance: a school-based study with objective monitoring of outdoor time
Source: BMC Public Health. 2023 Apr 4;23:645. doi: 10.1186/s12889-023-15532-y (PMC10071681; doi:10.1186/s12889-023-15532-y)
Supplement: Supplementary file 1 — Additional file 1: Appendix 1. Information about the wearable device. Appendix 2. Algorithm of outdoor/indoor discrimination. Appendix 3. Compliance. [file 12889_2023_15532_MOESM1_ESM.docx]

**List of appendix**

1. Information about the wearable device

2. Algorithm of outdoor/indoor discrimination

3. Compliance

**Appendix 1 Information about the wearable device**

The smart wearable designed and developed by research team was equipped with a light sensor, accelerometer and GPS receiver. The light sensor samples luminance and

ultraviolet intensity at 20-s intervals. Both the front and back of the smart watch have light sensors to detect whether it is being worn. The accelerometer consists of three axes that indicate the X, Y, and Z axes in space and through filtering, peak-valley detection, and removing interference, and finally converts these into counting steps.


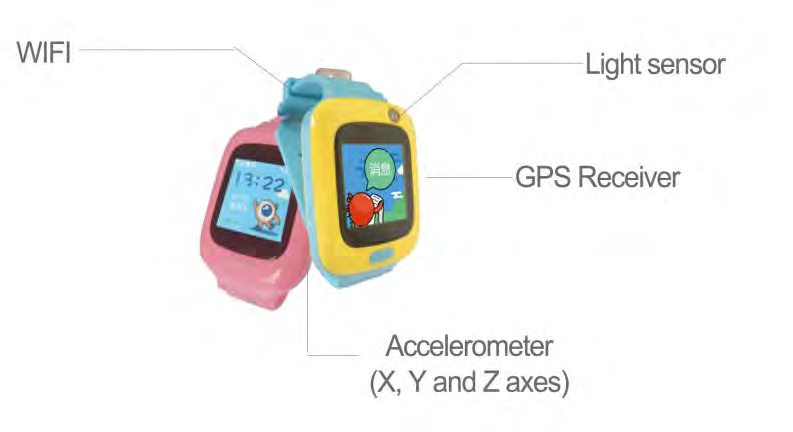


The built-in GPS receivers are used for receiving satellite signals and collecting data on the longitude and latitude of the location. Weather and temperature are synchronized in real time from the official website of the Shanghai Meteorological Bureau. The smart watch samples data once a minute. One piece of data consists of: time (year/month/day/00:00:00, three data points of luminance (lx), three data points on ultraviolet light intensity, count of steps, weather (sunny/cloudy) and wearing status. The above data were uploaded by the mobile terminal to a software platform, that was developed for collecting, analyzing, and counting the data.


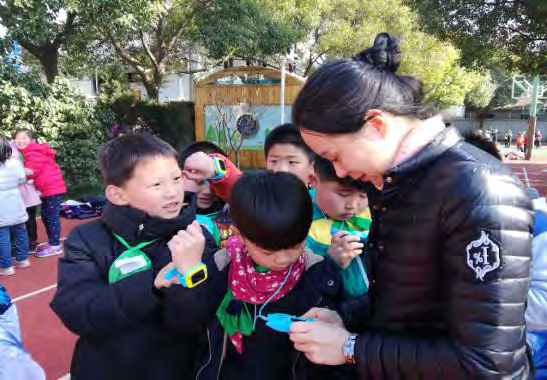


Consent was obtained from subjects in the picture.

**Appendix 2 Algorithm of outdoor/indoor discrimination**

Discrimination of environment to either an indoor or an outdoor environment could be converted into a binary classification problem. In machine learning, the computer learns a decision boundary in the feature space that separates or classifies the data points into two classes. When the training is completed, the learning is transferred to classify new data points based on the learned decision boundary. In binary classification, we chose support vector machine (SVM), as the tool to build the model due to its reported high accuracy. The model consists of the following variables: luminance, ultraviolet light levels and number of steps.

The core principle of the SVM algorithm is to establish a ‘hyperplane’ in the feature space that separates indoor and outdoor data by maximizing the distance between each of the data points from this hyperplane. In other words, firstly the algorithm involves finding the classification hyperplane. Thereafter, we adjusted the parameters which determined the hyperplane so that the distances from the data points to the separating hyperplane were maximized. Assuming we have ‘n’ points (xi, yi) in the training set, the parameters αi and b can define the hyperplane. The hyperplane can be formulated as following.

$$f(x)=\sum_{i=1}^{n} a_{i}y_{i}\left\langle x_{i},x \right\rangle+b$$

As the various data collected by smart watches are nonlinear, we added ‘kernel

function’ to the model. That is, through the spatial transformation of φ (generally

low-dimensional space is mapped to high-dimensional space x → φ (x)) to achieve

nonlinear separation. Then the hyperplane defined in the transformed space

(high-dimensional space) can be formulated as following.

$$f(x)=\sum_{i=1}^{n} a_{i}y_{i}\left\langle\emptyset(x_{i}),\emptyset(x) \right\rangle+b$$

**The detailed methodology published reference:**

Ye B, Liu K, Cao S, Sankaridurg P, Li W, Luan M, Zhang B, Zhu J, Zou H, Xu X, He X.

Discrimination of indoor versus outdoor environmental state with machine learning algorithms in

myopia observational studies[J]. Journal of Translational Medicine, 2019, 17(1): 314-325

**Appendix 3 Compliance**

Light sensors on the front and back of the smart watch could detect if it is worn. The data collected by the watch will be uploaded to the real-time monitoring information system within a certain time interval. And many people were arranged to supervise the usage of the wearable device and the records. Community leaders, head teachers and health care specialists reminded and supervised everyday. Besides, there were expert supervision committee, and supervisors from municipal and district level.
